# Barriers to mental health care utilization among internally displaced persons in the republic of Georgia: a rapid appraisal study

**DOI:** 10.1186/s12913-018-3113-y

**Published:** 2018-04-30

**Authors:** Adrianna Murphy, Ivdity Chikovani, Maia Uchaneishvili, Nino Makhashvili, Bayard Roberts

**Affiliations:** 1 London School of Hygiene and Tropical Medicine, Centre for Global Chronic Conditions, 15-17 Tavistock Place, London, WC1H 9SH UK; 2 Curatio International Foundation, 3 Kavsadze str., Office 5, 0179 Tbilisi, Georgia; 3 Ilia State University, 3/5, Kakuca Cholokashvili Ave, Tbilisi, Georgia

**Keywords:** Mental health services, Health systems, Low- and middle-income countries, Conflict

## Abstract

**Background:**

There is a paucity of evidence on access to services for mental health and psychosocial support for conflict-affected populations in low- and middle-income countries. In the Republic of Georgia, rates of utilization of mental health services among internally displaced people with mental disorders are low. We set out to identify the health system barriers leading to this treatment gap.

**Methods:**

We used rapid appraisal methods (collection and triangulation of multiple data sources) to investigate barriers to accessing mental health care services among adult IDPs in Georgia. Data collection included review of existing policy documents and other published data, as well as semi-structured interviews with 29 key informants including policy makers, NGO staff, health professionals and patients.

**Results:**

The following factors emerged as important barriers affecting access to mental health care services among IDPs in Georgia: inadequate insurance coverage of mental disorders and poor identification and referral systems, underfunding, shortage of human resources, poor information systems, patient out-of-pocket payments and stigmatization.

**Conclusion:**

While rapid appraisal methods cannot control for potential biases or achieve representativeness, triangulation supports internal validity and reliability of the data collected, allowing data to be used to inform health care interventions. The appropriateness and potential effectiveness of policy interventions such as insurance coverage of a wider range of mental disorders, integration of services for these at the primary health care level, and community-based approaches in this context should be explored.

**Electronic supplementary material:**

The online version of this article (10.1186/s12913-018-3113-y) contains supplementary material, which is available to authorized users.

## Background

Recent analyses of the global burden of disease attributable to mental disorders have called attention to the magnitude of this burden and the urgent need for improved systems of prevention and treatment of these diseases [1]. Yet health systems globally are failing to meet this need, [2] especially in low-and middle-income countries (LMICs) [3, 4]. Potential effects of untreated mental disorders include: personal suffering and distress, poor social functioning, lower productivity, and increased likelihood of physical illnesses – particularly chronic conditions for which treatment adherence may be impacted [5, 6]. While there is an increasing evidence base of cost-effective interventions for mental disorders, the majority of people with mental disorders in LMICs do not receive treatment [7]. Effective treatment of mental disorders may be especially challenging in LMICs currently or recently engaged in armed conflict, where the likelihood of mental disorders such as post-traumatic stress disorder, depression and anxiety is increased and health systems are destabilized [8–10]. Despite the well-documented high-levels of mental health needs among conflict-affected populations, [8, 9] there is much less evidence on access to services for mental health care services for conflict-affected persons in LMICs (which is where the vast majority of conflict-affected persons live), with only a few studies explicitly investigating this [11–15]. What research does exist from conflict-affected settings in the former Soviet Union region has revealed large gaps in treatment of mental disorders. For example, one study in Ukraine found that 74% of IDPs who likely required mental health and psycho-social support did not receive it [15]. Another study, from Kosovo, among female civilians 10 years after the war found that more than half used health care services during the previous three months but only a small minority used specialist mental health services [16]. There is also little evidence on the role of health systems in responding to the mental health care needs of conflict-affected populations, [17] despite its importance.

One conflict-affected population where research on mental health care access is needed is in the Republic of Georgia. Georgia has experienced two main phases of conflict, each of which involved secessionist movements [18, 19]. The first phase occurred in the early 1990s, in the regions of Abkhazia and Shida Qartli (South Ossetia), and led to the internal displacement of approximately 300,000 people, of whom approximately 200,000 remain as internally displaced persons (IDPs). The second phase occurred in August 2008, due to conflict between Georgia and the Russian Federation over Shida Qartli. This phase led to at least 128,000 ethnic Georgians being internally displaced, of whom up to 100,000 have now returned to their home areas. Some 200,000 Georgians therefore, remain as IDPs [20]. Approximately 40% of these live in collective centres, with the remaining IDPs in private accommodation. Collective centres are usually made up of former public or administrative buildings, or purpose-built villages constructed by the government after the 2008 conflict. These IDP communities are characterised by poor living conditions, high unemployment, poverty, limited integration with local communities and financial barriers to access health care and medicines. Recent estimates of the prevalence of mental health disorders among the IDP population found a persistent high burden of psychiatric symptoms and disability. For example, the estimated prevalence of post-traumatic stress disorder (PTSD) among IDPs in Georgia is 27.1% (1990s IDPs) and 22.9% (2008 IDPs); for depression these figures are 18.7 and 9.9% and for anxiety 13.0% and 9.2% [19] Despite this evident need for services however, research on utilization of mental health care among IDPs in Georgia found that only just over a third of those with a current mental disorder sought any assistance from health services. Approximately 27% did not seek care because they did not self-report having problems, while roughly 33% did report problems but cited barriers to accessing care, most commonly not being able to afford it [13].

Given the large treatment gap uncovered in Georgia among IDPs with symptoms of mental disorders, it is clear that barriers remain in access to treatment of mental disorders among IDPs, and that care users with mental disorders may not be accessing adequate care. The aim of this study is to explore how mental health care for IDPs is organized in Georgia, and to identify both user and provider-side barriers that may impede access to primary and secondary mental health care services for this population. As our data collection occurs five years after the most recent phase of conflict, we focus on access to routine mental health care services (i.e. those that are available to the general population) rather than designated mental health and psycho-social support (MHPSS) services that are usually provided in the immediate aftermath of a humanitarian emergency.

## Methods

This study used rapid appraisal (RA) methods to explore barriers to access to mental health care among adult IDPs in Georgia. The RA approach involves collection and triangulation of multiple data sources to provide an understanding of a situation in a more timely and cost-effective manner than standard social research methods, and to seek a diverse range of perspectives, without aiming for statistical precision [21]. Triangulation provides internal validity and reliability of the data collected, [22] and these data can then be used to develop specific health care interventions [21, 22]. Rapid appraisals of health systems have the advantage of requiring only limited time and resources while still providing reliable findings and have previously been successfully used for understanding health system performance for non-communicable diseases (NCDs) such as diabetes, [22, 23], including in Georgia [24, 25]. First, we undertook an initial review of the system for mental health care in Georgia using available documents. The document review involved examination of available policy documents, studies and evaluation reports [13, 18–20, 24, 26–31] with the aim of obtaining a comprehensive understanding of the organization and implementation of mental health care in the context of the national health system, and in particular the existing mental health service structure for IDPs in Georgia.

Then, to better understand how the mental health care system for IDPs works in practice and where the barriers may lie, we conducted semi-structured interviews with stakeholders from a range of perspectives - governmental and non-governmental actors, mental health care providers and mental health care users themselves. Participants from the first two groups were identified by snowball sampling i.e. identifying initial key stakeholders to invite as participants, who then provides the name of a subsequent participants, and so on.

Mental health care users were sampled from the Mtskheta-Mtianeti region, about 30 k north-west of Tbilisi and home to the Tserovani IDP settlement, the largest settlement of 2008 IDPs, as well as from the city of Gori, which was heavily affected by the 2008 conflict and is home to many IDPs living in collective centres. IDPs were sampled using convenience sampling (sampling based on accessibility and ease). Specifically, users were recruited by asking health professionals involved in the study to approach users who came in for mental health care and/or to call existing users by phone and invite them to participate in the study. This approach was chosen as it was more practical and likely to result in participation than attempting to identify participants with mental disorders in the community. Providers were asked to recruit users with a diverse set of mental disorders in order to capture various perspectives.

Our final sample included the following stakeholders:

i) Senior Ministry of Health Officials (*n* = 2);
ii) Directors from national and subnational NGOs (*n* = 4) that provided medical, psychosocial and legal support to IDPs in the aftermath of the conflict and with knowledge of the organization, financing and delivery of mental health care for IDPs;
iii) Health care professionals involved in both primary and secondary care of mental disorders: general practitioners (*n* = 3), neurologists (*n* = 2), psychiatrists (*n* = 3) and a home caregiver (*n* = 1); and
iv) IDP care users receiving medical treatment and psychosocial rehabilitation (e.g. cognitive behavioural therapy) for PTSD, depression and anxiety-phobic disorders from governmental and non-governmental facilities (*n* = 13).

The final number of key informants was *n* = 28. All interviews were conducted in Georgian using a semi-structured interview guide. Semi-structured topic guides allow for the interview to be participant-driven and are thus useful for exploration of an individual’s perception of a particular phenomenon [32]. Topic guides covered the burden of mental health care in Georgia, the organization of provision and financing of mental health care services for IDPs in Georgia, the user pathway, including diagnosis, treatment and follow-up, pharmaceuticals and communication of knowledge regarding mental health care to IDPs. The guides for each of the interview participant categories can be found in Additional files 1, 2 and 3.

Interviews were conducted in October 2013 in Tbilisi (the capital city of Georgia) and in Mtskheta-Mtianeti region. They were conducted in participants’ offices (in the case of government or NGO stakeholders and health professionals), or in a private room in the health facility (in the case of care users). Prior to inclusion in the study, all participants were given an information sheet and consent form in their native language. Each interview lasted approximately 60 min. Interviews were conducted by trained researchers from Curatio International Foundation (Tbilisi), who have experience conducting research with IDPs about sensitive issues. Interviews with care users were conducted in their homes, those with providers were conducted at their place of work. All interviews were audio-recorded, transcribed and translated into English by Curatio researchers. Interview data were then analysed using inductive thematic analysis methods, Inductive thematic analysis involves coding data for emerging themes and concepts without trying to accommodate a hypothetical framework, and is thus ‘data driven’ and reflexive [33, 34] Analysis followed the steps to thematic analysis outlined by Braun and Clarke [33] and emerging themes were triangulated with data from the above mentioned document review. All analyses were conducted using NVivo 10™.

## Results

### Overview of mental health care system for IDPs in Georgia

In 2013, the Georgian government introduced the Universal Health Coverage (UHC) Programme a state-funded programme providing health care insurance to all members of the population that are not covered by a private health insurance scheme and from 2017 whose income level is below defined level. Most services covered by the UHC program require some co-payment, except for target groups that include poor population, IDPs from the 2008 conflict living in collective centres and other groups who are exempt from co-payment. The UHC program does not include coverage for inpatient and out-patient mental health services, although offers coverage for management of mild depression at the primary care level.

Mental health care services are covered instead by the State Programme for Mental Health (SPMH), a programme introduced in 1995 and managed by the Health Care Department, as part of the Ministry of Labour, Health and Social Affairs [28]. The SPMH offers coverage to all Georgian citizens for inpatient and outpatient psychiatric services. Outpatient services covered by the SPMH include: i) consultations and prescriptions provided by psychiatrists in Primary Health Care (PHC) centres with integrated psychiatric services, separately standing psycho-neurologic dispensaries, or outpatient departments in psychiatric hospitals; ii) psycho-social rehabilitation services; and iii) psychiatric crisis resolution day-care beds. While PTSD is covered for outpatient services, there are a number of common mental disorders excluded from the list of covered outpatient services, including anxiety and obsessive compulsive disorders. Many of these disorders are covered at Crisis Management Centres, (centres that provide outpatient support to people experiencing an extreme mental health or emotional crisis), but the number and geographic distribution of these types of centres is limited, and disorders are only treated if symptoms are severe (e.g. in danger of harming oneself or others or requiring hospitalization). The list of disorders covered for by SMPH for outpatient care, and those covered by the Crisis Management Centres, is included as Additional file 4. With respect to inpatient services, the SMPH provides all inpatient services for mental disorders free of charge (i.e. no co-payment), except for psychoactive drugs related psychiatric disorders, which are only provided with a substantial co-payment. Table 1 outlines mental health services available in Georgia by service provider, facility and funding source [13]. Earlier quantitative research with IDPs found that being covered by the government general insurance scheme in place at the time increased the odds of health service utilization for emotional and behavioural problems (as did being female and aged 40 years or older), and that the most common reason reported for not seeking care was the inability to afford care or drugs [13]

**Table 1:** Mental health services available in Georgia by service provider, facility and funding source

Health care provider	Health care facility	Funding source	Mental Health services
Pharmacy	Retail drug store	Out-of-pocket payment	Drug selling; advice on drugs
GP	Primary Health Care (PHC) Center	UHC	Management of mild depression and prescription of antidepressants, (free outpatient MH drugs are not provided)
Neurologist	PHC center, outpatient ward of clinic	UHC	Management of mild depression and prescription of antidepressants (free MH medications are not provided).
Psychiatrist	PHC center, psycho-neurologic dispensary, outpatient ward of psychiatric clinic	SPMH	Outpatient care (defined list of mental disorders (see Additional files 4), counseling, free outpatient drugs provision
Psychiatrist	Mental health ward at general hospital, acute psychiatric department/ward	SPMH	Inpatient care (all mental disorders requiring an acute inpatient treatment), counseling, free medications.
Psychiatrist, Psychologist, Social worker	Psychosocial rehabilitation centres; Crises Management Centers; Mobile services	SPMH; Donor funds	Multidisciplinary case management (wide range of disorders, see Additional files 4), free outpatient drug provision; community mobile services
Psychiatrist, Psychologist, Psychotherapist	Private clinic	Out-of-pocket payment	Counseling, psychotherapy, drug therapy

*UHC* Universal Health Care Programme, *SMPH* State Programme for Mental Health

.

Approximately 2.8% of total government expenditure on health is devoted to mental health care (compared to 10.8% in the United Kingdom and 7.6% in Australia [35, 36]. Since 2006 there has been a gradual increase in funding (from 4.9 million GEL in 2006 to 10.7 million in 2011 and 16 million in 2016), but this is primarily allocated to inpatient services (70%). Only 0.44% of funds are used for psycho-social rehabilitation of individuals with mental health disorders [30].

According to state statistics on mental health, the rate of mental disorders per 100,000 population in Georgia in 2015 was 2682.5 [37]. (The reliability of this estimate is questioned, due to lack of surveillance data and some experts suggest the real rate of mental disorders may be two or more times higher [38]). Georgia has 9.9 psychiatrists per 100,000 population, 12.8 psychologists per 100,000 population, 7.7 nurses and 2.9 social workers. The average rates of these for the World Health Organization European region are 22.2, 45.3 and 60. There are nine psychiatric inpatient care facilities, two psychiatric inpatient wards in general hospitals in Georgia (there were three earlier but one was closed in January 2017) and 18 outpatient facilities (either integrated in PHC center or separately standing); 5 of these 18 are authorized to provide psycho-social rehabilitation counseling and four centers are providing crises management services [26, 30, 36, 37].

While there are no government services specifically targeted at IDPs, uninsured IDPs (as well as other citizens of Georgia) are eligible for benefits offered by the UHC programme and SMPH. The non-governmental sector was actively involved in health service provision (including mental health care service provision) to IDP populations in the immediate aftermath of the 2008 conflict in Georgia. Many international NGOs launched large-scale public mental health interventions targeting IDPs; however,these activities were criticized as poorly planned, superficial and culturally-insensitive, and unsuccessful in the short-and long-term [27]. Local NGOs provided services according to Inter-Agency Standing Committee guidelines, [39] recruiting field workers, training and supervising them and establishing referral pathways according to non-specialized and specialized care. Conflict-affected individuals were repeatedly screened after 1, 3, 6 and 12 months and interventions tailored accordingly. Local NGOs continued to provide evidence-based therapies (e.g. cognitive behavioural therapy) for disorders common among IDPs, including PTSD, depression and anxiety, but these activities have not been funded since 2016. The last externally funded project (supported by the EU commission) providing psychosocial rehabilitation services to IDPs in the Tserovani settlement specifically, ended in 2016. Georgian Church-supported activities for IDPs in Gori also ended in 2016. Currently there are no mental health services targeting to IDPs funded from NGO sources.

### Barriers to accessing mental health care among IDPs identified in interviews

Through our document review and interviews we identified several aspects of mental health care in Georgia that may explain low utilization rates among IDPs. We have presented these barriers below.

### Lack of comprehensive insurance coverage, case identification and referral system

A prominent theme that emerged in our key informant interviews is the lack of comprehensiveness of insurance coverage (i.e. what treatments are covered by insurance or not) offered by the SPMH, which may impact on effective identification and referral of patients with some mental disorders. Under the SMPH, major disorders such as depression, PTSD and schizophrenia are covered, but other disorders, such as anxiety-phobic disorders, are not covered. The prevalence of anxiety among IDPs in Georgia is estimated at 13.0% (1990s IDPs) and 9.2% (2008 IDPs)) [19] and the proportion of those self-reporting anxiety and not using care is high (21.6%) [13]. The SMPH does not prioritize prevention, and in particular screening, for any mental disorder, and resources to conduct such screenings are limited. As indicated by the director of a national mental health NGO:

*“The mental health program is quite inadequate not only in terms of displaced people but also in terms of prisoners as well because the State program only provides coverage for psychotic disorders. The diagnostics provided by the State program are inadequate”.*

And by that of a Tbilisi psychiatrist:

*“The family physicians have the ability to screen for and detect mental disorders, but in the reality they are not trained and these activities are not undertaken and referrals are rarely made. In fact, they (users) are referred mostly based on personal acquaintances. Even in our centre, which, in my opinion, works in full accordance with European standards and is dramatically different from other service providers; also here the referrals take place on the basis of personal acquaintances.”*

### Limited funding for mental health care

Limited funding emerged as a major challenge to effectively providing health services, both with respect to securing an adequate government budget for mental health care and to providing facilities with sufficient resources to implement SPMH guidelines. The former was highlighted in an interview with a high-ranking official at the Ministry of Health.

*“Generally I am very skeptical about the ability of the Ministry to fund something for two reasons: the first reason is that the country’s budget is entirely in deficit here and we have the same problem which was in 90-ies; that means we do not have enough amount of money to meet our requirements, the second problems implies that it is not an interesting subject in a political way and this issue had never been interesting for the Ministry.”*

The challenges facing providers at under-funded facilities were also emphasised in the interviews with providers:

*“If funding is not increased, it is impossible to follow the guidelines.” (Psychiatrist Gori)*

*“The new guideline was not followed by training yet…But the trainings are of no great use without the appropriate funding.” (Psychiatrist Tbilisi)*

There were also concerns about the way in which funding is allocated to the SMPH and how facilities allocate this funding to different services. The budget for the SMPH is allocated on the basis of historical budgets, which are proposed by the service providers but are based on unreliable data on the demand and utilisation of various services. Key informants in our study expressed concern that facilities may focus their spending on individual aspects of mental health care instead of providing a comprehensive service. As expressed by a senior health services officer at the Ministry of Health:

*“Legally, they do not have the right to do so (to allocate funds ad hoc to different services), because they provide the service in the specific component and are supposed to spend the pre-determined among of funds for this component on the beneficiaries of this component. Yet, this is what is happening, because sometimes funds are not enough in one component, afterwards it is not enough in another component.”*

### Poor human resource capacity in mental health field

Our interviews also highlighted an overall shortage of staff working in the field of mental health, in terms of both psychiatrists and psychiatric nurses, for example as experienced by a patient:

“*The queues! I cannot reach the doctor, as she has too many patients and is only one. I often go at 6 am in the summer - now in the winter it’s too cold to bear. There are always so many people that you should reserve a place in a queue in advance, otherwise you won’t get seen later.”*

This shortage might be partly contributed to by lower pay compared to other specialties, which deters doctors or nurses from specialising in psychiatry. However, some interviews suggested that perhaps rather than a shortage of trained professionals, it is an unequal distribution of human resources among institutions treating mental disorders that may present the largest barrier to effectively providing mental health care. As described by a senior health official from the Ministry of Health:

*The average salary of a psychiatrist is 240 GEL (approximately 90 USD) and that is the lowest salary in the health sector. A family doctor makes 3 times more than a psychiatrist.*

*There is a shortage - we have a lack of certain staff and psychiatrists. We have a lack of psychiatric nurses, of which I’m not sure we have any at all, and a lack of psychologists, psychotherapists and social workers. It sounds very negative when I speak about it, but some institutions are very overcrowded with medical staff; for example, there are the institutions outside of Tbilisi, where there is a lack of the staff and there are the institutions in Tbilisi, where there are five hundred people in one institution. There is very big difference when it comes to staff distribution (between central and regional providers). For example, as I know, in Qutiri, 700 users are served by three or four psychiatrists and the rest are mostly nurses. As another example, in 2009, we wanted to send a psychiatrist to Oni district at the request of a governor, but we could not physically find a specialist to send there.*

Moreover, our interviews emphasized a low level of knowledge and skills required to diagnose and manage people with mild mental health illnesses, especially in primary care settings. Particularly relevant to IDPs, it appears that even among the trained staff that does exist, there may be a shortage of specialists who can diagnose and manage PTSD. Referral of IDP mental health care users by primary care physicians to neurologists instead of psychiatrists seems common, but may be due to the stigma associated with mental disorders and a lack of clear guidelines for diagnosis and treatment of conditions such as PTSD and anxiety, as well a lack of training in the symptoms of these disorders. This seems to result in users only seeing a psychiatrist at advanced stages of their disorder. Two quotes that illustrate this are the following:

*Today, virtually nothing is happening as it should. The family doctor, when visited by a patient with symptoms of anxiety, refers the patient to the neurologist, which in my opinion is not correct.*

*Skills are low – especially in the periphery, in some cases, doctors (psychiatrists) find it difficult to take responsibility and instead send the patients to Tbilisi in order to get a diagnosis confirmed and treatment regime defined. Only then does the doctor feel comfortable prescribing medication on a monthly basis (Senior Health Services Officer, Ministry of Health)*

*“Psychiatrists even are not making the diagnosis of post-traumatic stress disorder. There are no specialists of neurotic disorders in Georgia. The role of neurologists is completely inadequate because they try to handle disorders which absolutely are not within their competence. By mandate, these disorders are not included in neurology, so I really don’t know why they are doing this.” (National Mental Health NGO Director)*

And from psychiatrists themselves:

*“There are many cases when our patient is referred to the neurologist and after a long time they come to us with advanced disorders.” (Psychiatrist, Gori)*

*“There are some guidelines in psychiatry; however, majority of psychiatrists still use the old methods when treating patients.” (Psychiatrist, Mtskheta)*

### Absence of high-quality information systems

A further barrier to the successful delivery of mental health care to IDPs that was emphasized by our interviews is the absence of high-quality information systems to register and maintain data on IDP patients. The lack of high quality and reliable data on population need prevents effective planning of mental health care delivery for this population. Data are only available for those who are registered in the SMPH, resulting in a likely underestimate of the burden of and range of mental health disorders among IDPs in Georgia. Moreover, data that are collected on IDP mental health care users are likely to be unreliable. This lack of data appears to manifest itself at the local level as well, as GPs demonstrate low awareness of the burden of mental disorders among their patient population.

As described in the interview with a service official at the Ministry of Health:

*“We only receive data from clinics and agencies that are involved in the state programme. Data on cases and service provision from institutions that are funded by donors are not captured by our statistics. Also, in 2008-2009, registration of patients was possibly without an ID card and it's unclear whether patients registered with their real names or with fake names. The reasons for each visit are also unclear - for example, I don't know how these 80,000 visits got into the statistics or what disorders they were for.”*

*“Mental disorder cases are not frequent in Georgia, though they exist. At least I think they are not frequent.” (General Practitioner, Gori )*

*“I cannot tell you what the need (for mental health care) is here. I have no statistical information about how many mental health care users live in Gori.” (General Practitioner, Gori)*

### Absence of political will to enact reform

Several stakeholder interviews highlighted the lack of recognition of the particular mental health needs of IDPs and the political will to enact reform that would recognize these needs. There is no specific government programme or policy targeted at the mental health needs of IDPs, and no resources have been allocated specifically for mental health care for IDPs. The following quotation from the director of a national NGO providing services to IDPs was one example highlighting this issue:

*“There is no work (on mental health care needs of IDPs). These people are absolutely neglected. This problem isn’t even considered by the government; there is absolute silence about IDPs. There wasn’t even one story concerning the IDPs of 2008 and the society doesn’t know about them.”*

Our interviews also shed light on the failure of NGOs and the SPMH to co-operate with each other toward the effective provision of mental health care to vulnerable populations. Due to the lack of coverage for certain mental disorders prevalent among IDPs, NGOs play a prominent role in providing care and medication this population. These NGOs seem to have very limited association with the SPMH in terms of streamlining provision of mental health care provision. This may be mainly because the state programme does not include the particular mental health needs of the IDP populations treated by NGOs, and care users often prefer to use the services provided by these NGOs than the state services. As pointed out by the director of one regional mental health NGO:

*“The Ministry of health doesn’t work in the context of IDPs, only in the context of former prisoners…At first they seemed interested in IDPs, we wrote a budget proposal for IDP mental health care for them…We wrote the text and then they disappeared.”*

#### Micro-level barriers

Our interviews highlighted several micro-level, or user-level, barriers to accessing effective mental health care among IDPs. These barriers may be, and often likely are, downstream effects of elements of the national mental health care system, but we classify them here as micro-level barriers because they are experienced (and therefore usually reported) at the user-level rather than by government officials or providers.

### Out-of-pocket costs incurred for mental health care

Out-of-pocket expenses for consultations and medications were commonly reported among IDP mental health care users. While in many cases this may be because some disorders experienced are not covered by the SPMH, in other cases users seem to be completely unaware of the existence of the SPMH and the services to which they are entitled free-of-charge. Users are only eligible for coverage of consultations and medications if they are registered in the SPMH.

*Interviewer: What about those medicines prescribed by the neurologist? Did you buy them yourself?*

*User: Yes, yes,*

*Interviewer - Nothing for free?*

*User: No*

*Interviewer: Did you pay for the consultations provided?*

*User: Sure, I even paid for the consultations by the psychiatrist*

Out-of-pocket costs for consultations may impact on health-care seeking decisions. In order to avoid the cost of a review consultation, clinic users simply continue their medication by purchasing directly from the pharmacies. The cost of medications is also not fixed, which may affect user decisions to purchase medications consistently.

*Interviewer - When you need Troxane, do you buy it does the doctor give it to you?*

*User - When I visit the doctor, he gives me 10 tablet for free, but he doesn’t provide any prescription for further purchasing. If I have to pay for the visit, then I prefer to buy the medicine myself because 100 Tablets cost 7 GEL. It is better than to pay for doctor's visit and then get the medicine for free… I prefer to buy it in pharmacy, because I have to pay in transport, then for consultation, so the pharmacy is the best option.*

*Interviewer – Does the doctor give you prescriptions for any of the other medicines?*

*User - As for Dozylen, yes. It is quite expensive though and it’s price changes. Once I paied 14 GEL for 24 tablets, now we pay 20 GEL.*

The SPMH does not cover the cost of consultations and treatment given by neurologists. Given that many mental health care users are referred to and managed by neurologists for a long duration, many users would have paid a substantial amount of out-of-pocket expense on these consultations and medications prescribed.

### Low mental health awareness and stigma associated with mental disorders

From our interviews, stigma emerged as a prevalent factor in user behavior. Stigma associated with mental disorders in Georgia may delay users being diagnosed and receiving appropriate treatment (due to shame associated with seeing a psychiatrist), or may result in their paying for treatment unnecessarily (due to a reluctance to be enrolled in the SPMH or a preference to travel far from home to access treatment). The impact of stigma associated with mental disorders on accessing effective treatment for mental disorders is illustrated in quotations from our interviews with health care professionals in Mtskheta:

*“It is often that a user should be treated by a psychiatrist rather than a neurologist, but the parents are reluctant to take themselves or their child directly to the psychiatrist.” (Neurologist, Mtskheta)*

*“Due to the stigma, users try to the bitter end not to go to a psychiatrist. Depending on his/her social status, he/she begins to take an alternative treatment (fortune-tellers, mullahs). If there were a psychiatrist on staff at a polyclinic (as opposed to only at psychiatric clinics), this may improve the chances that patients will go see them.” (Psychiatrist, Tbilisi)*

*“There are patients who are eligible to get pensions, but do not get registered and visit the doctor to receive a free consultation. They prefer to self-prescribe and not disclose their disorder.” (General Practitioner, Mtskheta)*

*“I think stigma gets in the way of treatment. There was such a case last week: a very agitated person, a young man. He was talking roughly and became very nervous when I asked him about the amnesia: “Am I being interrogated here or what?!” he exclaimed, and then his mother asked us to treat him without registration. But I refused to provide such treatment and they went to the general clinic where they had been treated before. Many parents come here in advance and say: “Please, do not say (to the patient) that you are a psychiatrist, just pretend to be a neurologist” .Or, the patients’ parents or other family members come to get the drugs alone because the patients are ashamed of their condition.” (Psychiatrist, Mtskheta)*

## Discussion

The rate of mental health care utilisation among conflict-affected IDPs in Georgia is low. This paper is the first to use qualitative methods to provide insight into some of the barriers that may affect access to treatment and thus utilization rates in this population. The barriers that emerged as important appear consistent between patients and providers and other stakeholders, but experienced in different ways at different levels of the health system. Lack of comprehensive insurance coverage, case identification and referral of patients with mental disorders (resulting in out-of-pocket payments), and poor funding for mental health services emerged as two key system barriers to effective access to treatment for mental disorders in Georgia. While some of these barriers, in particular out-of-pocket payments for mental health care and medicines, have also been observed among the general population of Georgia, [29] it is likely that these barriers disproportionately affect IDPs given the high prevalence of mental disorders in the Georgian IDP population, and the generally low socio-economic status of this group. Other research suggests that stigma as a barrier to accessing care may be particularly relevant in IDP or refugee communities due to a history of fear and repression [40].

These barriers also reflect those observed with non-conflict-affected populations globally and in the region, related to under-investment in mental health financing, staffing, and facilities, and the way in which services are organized [5, 41–49]. The WHO has developed guidelines for policy-makers to support adequate financing of mental health care, stating that where possible, coverage of mental health services should be mandatory, through national, tax-based or social insurance [50]. These guidelines also identify factors that can result in government underfunding of mental health care, including inadequate recognition of the importance of mental disorders and their consequences for those who suffer from them and their families, and the failure of policy-makers to understand the potential effectiveness of interventions for mental disorders, resulting in an assumption that other services are more beneficial to the population [50]. The extent to which these factors are relevant in the Georgian context requires further investigation.

While, coverage, funding, human resources and other structural (or macro) barriers appear important, attitudinal barriers, specifically stigma associated with mental disorders, also plays a key role in preventing access to appropriate treatment. Our results are not dissimilar to results of World Mental Health Survey from 17 countries which showed that although structural barriers to accessing effective treatment were more prevalent among severe cases of mental disorders, attitudinal barriers were more important to initiating and continuing treatment for mild-moderate cases of mental ill health [51]. They are also not dissimilar from recent research on IDPs in Ukraine, where in addition to not being able to afford care and low awareness, trust or geographic access to high-quality services, stigma and embarrassment were found to be important barriers to using MHPSS [15].

One possible strategy for addressing these barriers is the integration of mental health care for a wider range of disorders, including anxiety and depression, into PHC services, with capacity-building of PHC personnel. There is evidence that integration of mental health care into PHC is cost-effective, improves access to care and reduces stigma and discrimination [52]. Integration is recommended by WHO [53] and a programme of integration of a basic package of mental heatlh care services into PHC is currently being evaluated in five low- and middle-income countries by the PRIME study [7]. Another approach for which there is some evidence of effectiveness in conflict or post-conflict settings creating new health worker cadres to fill service gaps. Health workers recruited from local communities can ensure long term retention and encourage culturally appropriate and acceptable interventions, [17] thus addressing human resource and stigma barriers. This approach could also address our finding of a low level of knowledge and skills required to diagnose and manage people with mild mental health illnesses, especially in primary care settings. Opportunities for reducing stigma might also include community-based approaches such as those advocated by UNHCR, that engage mental health care users and communities in promoting their own health and well-being, and use participatory assessment (dialogues with affected persons to understand needs and resources, and develop strategies to better address needs), to identify groups that need targeted attention [54]. The WHO and its partner agencies have also produced guidelines for low-intensity interventions aimed reducing symptoms of common mental disorders through behavioural strategies, and that can be delivered by laypeople from the affected communities, without formal mental health training [55]. Whether or not such approaches would be appropriate in the Georgian context requires investigation.

Our study has also contributed to the growing body of work demonstrating the potential value of RA as a tool understanding barriers to mental health treatment among conflict-affected populations [56–60]. Importantly, the study has taken a health systems approach, investigating potential barriers at all levels of the system, from government to providers and service users. Guidelines for mental health care assessment from the Inter-Agency Standing Committee recommend collection of health systems information for a comprehensive overview of mental health care, [39] RAs of barriers at various levels of the health system are rare. We have shown that with few resources and within a short time frame, RA methodology can provide a comprehensive overview of health system barriers to effective treatment of mental disorders among conflict-affected populations. In the early stages of conflict, RA might provide a useful tool for understanding mental health needs and health system barriers to care sooner, allowing for these to be addressed in a more timely manner than usual.

### Limitations

There are limitations to our methods that must be acknowledged. While we strove to include a diverse sample of participants, RA cannot provide representative data on barriers experienced and does not allow for controlling potential biases. This is particularly true in our case as we only interviewed existing users of mental health care services, rather than other community members who may have needed these services but did not access them. While we did so because it was more practical to identify individuals with mental disorders in this way, rather than among the general population, we may have either failed to identify some important barriers that were not experienced by those included in our study, or have overestimated the importance of the barriers that were identified. Similarly, we also did not interview family members of users of mental health services and they may have offered valuable additional insights into health care seeking behavior and attitudes.

## Conclusion

Inadequate insurance coverage, case identification and referral to treatment for mental disorders, underfunding, shortage of human resources, poor information systems, patient out-of-pocket payments and stigmatization all appear to be important factors affecting effective provision of mental health care services to IDPs in Georgia. The appropriateness and potential effectiveness of policy interventions such as insurance coverage of a wider range of mental disorders, integration of services for these at the primary health care level, and community-based approaches in this context should be explored.

## Additional files

Additional file 1: Topic guide for key informant interviews. (DOCX 13 kb)

Additional file 2: Topic guide for health professionals. (DOCX 14 kb)

Additional file 3: Topic Guide for IDPs with mental disorders. (DOCX 13 kb)

Additional file 4: Mental disorders covered by SMPH outpatient care. (DOCX 12 kb)

## Abbreviations

IDP: Internally displaced person
LMIC: low- and middle-income countries
MHPSS: Mental Health and Psychosocial Support
NCD: non-communicable disease
RA: Rapid Appraisal
SMPH: State Programme for Mental Health
UHC: Universal Health Care Programme
UNHCR: United Nations High Commissioner for Refugees
WHO: World Health Organization
