# Supplementary material for: Barriers to mental health care utilization among internally displaced persons in the republic of Georgia: a rapid appraisal study
Source: BMC Health Serv Res. 2018 Apr 30;18:306. doi: 10.1186/s12913-018-3113-y (PMC5928589; doi:10.1186/s12913-018-3113-y)
Supplement: Supplementary file 1 — Topic guide for key informant interviews. (DOCX 13 kb) [file 12913_2018_3113_MOESM1_ESM.docx]

## Appendix A: Topic guide for key informant interviews

**Please fill out the following:**

**Location __________________________________**

**Type of institution/health facility (MoH, PHC, hospital...)__________________________________**

**State/ private __________________________________**

**Size (what area does it serve (region, national…) __________________________________**

**Position of respondent (head of department, etc.) __________________________________**

**Respondent's specialty (GP, psychiatrist …) __________________________________**

**Sex __________________________________**

**Length of work experience at the above position ____________________________**

*Instruction:*

*This is a topic guide outlining the areas we want to understand. Not all KI will be informed about all these areas or will have the time to answer. Some KI will be from outside the health sector and only a few of the questions will be relevant. Below are some leading questions and follow-up probes but KI may follow their own line of thinking and may not like to be interrupted. They should be given opportunity to talk about issues where they have most expertise and personal interest, with some directing from the interviewer. Before each interview, think about what is the role of the respective KI and what are the several key things you want to get out of them.*

**Introduction:**

*Interviewer:* Hello my name is _____________. Thank you very much for agreeing to participate in this interview today. As you have been told, your interview will be recorded but your identity will be kept confidential so you should be encouraged to speak freely. You are also free to stop the interview at any time or refuse to answer any questions.

1. Could you tell us about yourself and your organization and its relationship to mental health among IDPs.

2. Could you talk about the prevalence of mental disorders among IDPs in Georgia? (morbidity, mortality, who is affected, common and severe complications)

3. To what extent is mental health among IDPs specifically a health priority in Georgia?

4. Could you describe the legislation, strategies and regulations directly or indirectly relevant to mental health care among the general population and among IDPs? (within or outside the health system)

5. What are your views on the way prevention of mental disorders works for IDPs in Georgia? (programmes, institutions involved, target groups, mechanisms…)

6. Who is involved in responding to mental disorders – within and outside the health system?

7. Could you assess the existing physical infrastructure, human resources and capacity in mental health care for IDPs? To what extent each of these are adequate given the need?

8. Entry into the health system: How do mental health care users get diagnosed usually?

9. Treatment and follow-up: Could you now tell us how do users move through the system, once diagnosed?

10. What are the most common risk factors for mental disorders? Are there any groups that are more at risk, and why?

11. Best practice: What are the requirements for effective management of mental disorders? What constitutes best practice? Are there any constraints to practicing this in reality, especially among IDPs?

12. How is mental health care financed? (drugs, staff costs, infrastructure)?

13. Assess the way procurement and distribution of drugs for mental disorders are organized in Georgia.

14. Communication: What is the awareness among the IDP population, mental disorders sufferers and health staff regarding mental disorders and its treatment?

15. Information: what information is available on mental health? On mental health of IDPs specifically?
