# Supplementary material for: Barriers to mental health care utilization among internally displaced persons in the republic of Georgia: a rapid appraisal study
Source: BMC Health Serv Res. 2018 Apr 30;18:306. doi: 10.1186/s12913-018-3113-y (PMC5928589; doi:10.1186/s12913-018-3113-y)
Supplement: Supplementary file 2 — Topic guide for health professionals. (DOCX 14 kb) [file 12913_2018_3113_MOESM2_ESM.docx]

## Appendix B: Topic guide for health professionals

**Please fill out the following:**

**Location __________________________________**

**Type of institution/health facility (MoH, PHC, hospital...)__________________________________**

**State/ private __________________________________**

**Size (what area does it serve (region, national…) __________________________________**

**Position of respondent (head of department, etc.) __________________________________**

**Respondent's specialty (GP, psychiatrist …) __________________________________**

**Sex __________________________________**

**Length of work experience at the above position ____________________________**

*Instruction:*

*Please do not ask all questions in the given order, but decide which questions to ask and in what order according to the circumstances of the particular person. The idea is to allow respondents first to talk about their experience following their own logic and to feel more relaxed with the interview process. You could use probes (usually given in brackets) to lead the conversation in a particular direction, or reminding them of things they have said earlier in the conversation. Do not directly read probes to the respondent, first give them time to answer the question in their own words. Key themes/questions are given in bold.*

**1. Burden of mental disorders.**

• Do you think there is a lot of mental disorders in Georgia? What are the possible reasons?

**2. Mental health as a health priority.**

• Is addressing mental disorders among IDPs a priority currently given the current morbidity and health needs in Georgia?

**3. Assessment of legislative and regulatory framework**

• Could you describe the main regulations with any relevance to mental health care that is relevant to your work?

**4. Prevention/Diagnosis**

• How effective is screening as a strategy to identify mental disorders in IDPs? (discuss strategies: population-based approaches, targeting high-risk groups)

**5. Management of mental disorders: institutional arrangements and roles**

• What institutions or health care professionals are involved in management of mental disorders among IDPs? • Which ones should be involved, in your view, and in what capacity?

**6. Infrastructure and capacity**

• In your institution are there any activities regarding mental disorders? Describe your role in this.

• How many IDPs with mental disorders do you see usually per month? Can you talk a little about the population you cover, the resources at your disposal and any obstacles your institution faces?

**7. Pathways to care**

• Usually how is mental disorders diagnosed among IDPs (and/or generally)? [screening for mental disorders/other checkups/ if symptoms; initiative of people, referred from elsewhere]

• Do people face any barriers in access to diagnostic care? • How could early detection of mental disorders be facilitated?

**8. Treatment**

• Once users are diagnosed, describe what happens.

• Are there any cases where this sequence is different? Give examples. Why?

• Do IDPs face any barriers in treatment for mental disorders?

• Are there any ways that you think treatment of mental disorders among IDPs could be improved?

**9. Follow-up and linkages**

• Do IDP mental health care users have a designated health care professional that is mainly looking after them?

• Are there any obstacles to successful management of mental disorders? Are they related to the user, the health system, social and economic background? Discuss each of these.

**10.** **Funding**

• How is mental health care for IDPs financed?

• Are there any financial incentives to treat mental disorders among IDPs?

**11. Pharmaceuticals**

**•** How are pharmaceuticals financed in Georgia? • Are there shortages in drugs and consumables? Could all IDP care users with mental disorders obtain all necessary drugs, the right brand, at the right time or are there problems?

• Is medicine generally prescribed properly? Used properly by the care users? Do people self-medicate?

• What kind of (insert name of drug) is available (brand names vs. generics)? What brands vs. generics? Imported from where?

• Could you describe how the pharmaceutical supply in your facility works? How do you request drugs, from whom, and how often? Who is responsible for distribution?

• What could be done to facilitate access to drugs, especially among IDP populations?

**12. Communication**

**•** To what extent there is an awareness among the IDP population regarding the risk factors for mental disorder and its symptoms?

• In your experience, how do mental health care users (already diagnosed) find out about mental disorders? (sources: health staff media, mass public health campaigns,…]

• Are there education programs for people with mental disorders? Who organizes these? Who is involved? Do people have to pay to be enrolled?

• How could awareness be improved?

**13.Information**

**•** Is there a national mental disorder registry? Is there a registry for IDPs specifically? How is information being filled and updated? Could you assess the data quality in it?

• Do you know how many people (IDPs) with mental disorders live in your catchment area?

**14. Concluding remarks**

• Do you think there is a potential for improvement of a) diagnosis; and b) continuous management of mental disorders? What could be done in your view?
