# Supplementary material for: Barriers to mental health care utilization among internally displaced persons in the republic of Georgia: a rapid appraisal study
Source: BMC Health Serv Res. 2018 Apr 30;18:306. doi: 10.1186/s12913-018-3113-y (PMC5928589; doi:10.1186/s12913-018-3113-y)
Supplement: Supplementary file 3 — Topic Guide for IDPs with mental disorders. (DOCX 13 kb) [file 12913_2018_3113_MOESM3_ESM.docx]

## Appendix C: Topic Guide for IDPs with mental disorders

*Instruction:*

Please do not ask all questions in the given order, but decide which questions to ask and in what order according to the circumstances of the particular person. The idea is to allow respondents first to talk about their experience following their own logic and to feel more relaxed with the interview process. You could use probes (usually given in brackets) to lead the conversation in a particular direction, or reminding them things they have said earlier in the conversation. Do not directly read probes to the respondent, first give them time to answer the question in their own words. Key questions are given in bold.

**Preliminary conversation, creating rapport, introductions.**

**Please fill out the following:**

**Age __________________________________**

**Sex __________________________________**

**IDP status __________________________________**

**Residence________________________________**

**Type of mental disorder ____________________________**

**Reason for seeking care________________________________**

**1. Onset of illness and diagnosis**

• Could you describe step-by-step all the contacts with the health system preceding the final diagnosis. For each contact, describe where it took place (location & type of facility), when, what tests/ treatment was done, who did it etc.

• Can we talk about your experience of this process? Did you face any difficulties during this process? Please discuss these.

• What helped you through this period? [e.g. health professionals, other people, institutions, financial support, social support]

**2. Treatment**

• Again, could we talk about what happened step by step, from diagnosis until now. Where did you go, who treated you, for how long, as far as you could remember.

• If any, what difficulties did you face during this process? [related to the health system.]

• Who is monitoring your illness? Where?

• Have you ever consulted a psychologist/psychiatrist? Otherwise who advises you on your disease management?

• If you have ever required emergency care, how did you get to the health facility? Do you know to get there, if you haven’t?

• Would you say it was easy or difficult to follow the prescribed treatment?

**3. Access to and use of applicable medicine.**

• Usually, where do you get your medicine?

• What kind of medicine do you use? Where is it produced? What brands/ or generic?

• Do you have to pay for it? If yes, usually how much per month?

• Are there shortages of medicine? Could all users with mental disorders obtain all necessary drugs, the right brand, always on time? Discuss problems.

• Have you ever had delays in obtaining medicine? Have you had times where you have to go without medicine for quite some time? [how long, what was the case]

• Do you get help from anybody in getting the medicine? [health worker, family, friends; financially/ and physically getting it]

**4. Linkages and Communication**

• Have you got a particular doctor or health professional who is mainly looking after you and who knows you well?

• Currently, what other people are involved in taking care of you? Do you always see the same people?

**5. Knowledge**

• To what extent have you been kept informed about you treatment? By whom? Did you understand at the time what is happening at each stage? Do you understand it better now? Are there things that you still don’t understand?

**6. General Assessment**

• From your experience what could be done to make life of people suffering from mental disorders easier? [in prevention, in diagnosis, in treatment]

• Are there any changes that need to be made outside the health care system?
