# Supplementary material for: Barriers to mental health care utilization among internally displaced persons in the republic of Georgia: a rapid appraisal study
Source: BMC Health Serv Res. 2018 Apr 30;18:306. doi: 10.1186/s12913-018-3113-y (PMC5928589; doi:10.1186/s12913-018-3113-y)
Supplement: Supplementary file 4 — Mental disorders covered by SMPH outpatient care. (DOCX 12 kb) [file 12913_2018_3113_MOESM4_ESM.docx]

**Appendix D: Mental disorders covered by SMPH outpatient care**

| **Code(ICD-10)** | **Disorders** |
| --- | --- |
| F00-F09 | Disorder of organic nature, including symptomatic disorder |
| F20-F29 | Schizophrenia, Schizoid disorders |
| F30-F39 | Affective disorders |
| *Among them F32* | *Mild depressive episode* |
| *Among them F33* | *Recurrent depressive disorder* |
| F43 | Acute stress reaction and adaptation disorder |
| *Among them F43.1* | *Post traumatic stress disorder PTSD* |
| F70-F79 | Mental retardation |
| F80-F89 | Disorders of psychological development |
| F90-F98 | Behavioural and emotional disorders with onset usually occurring in childhood and adolescence |

Mental disorders covered by Crisis Management Centres

| **Code(ICD-10)** | **Disorders** |
| --- | --- |
| F20 | Schizophrenia |
| F21 | Schizoid disorders |
| F23 | Brief psychotic disorder |
| F24 | Shared psychotic disorder |
| F25 | Achizoaffective disorders |
| F31 | Bipolar affective disorder |
| F32 | Depressive episode |
| F33 | Recurrent depressive disorder |
| F40 | Anxiety Phobic Disroder |
| F41 | Other Anxiety Disorder |
| F42 | Obsessive Compulsory Disorder |
| F43 | Acute stress reaction and adaptation disorder |
| F60 | Spesific personal disorders |
